# Early–Late Correlations of Growth Traits of *Eucalyptus urophylla* S.T. Blake Clones over a Rotation

**DOI:** 10.3390/plants14243725

**Published:** 2025-12-06

**Authors:** Jianchao Yin, Guangyou Li, Zhaohua Lu

**Affiliations:** Research Institute of Tropical Forestry, Chinese Academy of Forestry, Guangzhou 510520, China; yinjianchao2023@163.com (J.Y.); lgy@caf.ac.cn (G.L.)

**Keywords:** *Eucalyptus urophylla*, clone, early–late correlation, optimal selection age

## Abstract

*Eucalyptus urophylla* is a core tree species for short-rotation industrial timber plantations in South and Southwest China. However, the dynamic correlation rules of its growth traits during the full rotation period remain unclear, and the theoretical research on early selection is insufficient. In this study, 12 pure *E. urophylla* clones (including U6 and MLA as controls) were used as plant materials. Based on the data of tree height (H), diameter at breast height (DBH, D), and individual tree volume (V) from 0.5 to 7.5 years old, the correlation rules of early and late growth traits were explored, core predictive traits were screened, and the optimal selection age was determined through rank correlation, phenotypic and genetic correlation analyses, combined with regression modeling and selection efficiency calculation. Early selection of *E. urophylla* clones was feasible: after 3.5 years, the early–late phenotypic and genetic correlation coefficients of H, D, and V all reached significant or highly significant levels, and the genetic correlation coefficients were greater than the phenotypic ones, indicating that genetic factors dominated trait correlations with little environmental interference. All five established early selection regression models passed the highly significant test. Among them, the models of D-early versus D-late, V-early versus V-late, and D-early versus V-late had the highest coefficients of determination (0.9293–0.9385), making them the optimal selection traits; the models of H-early versus H-late and H-early versus V-late had lower coefficients of determination (0.8010–0.8364) due to errors in height measurement. The best selection effect was achieved within 1/2–2/3 of the rotation period: for a 6-year rotation period (pulpwood), the optimal selection age was 3.5 years old (annual efficiency 1.318); for an 8-year rotation period (medium-diameter timber), it was 4.5 years old (annual efficiency 1.345); and for a 12-year rotation period (large-diameter timber), it was 6.5 years old (annual efficiency 1.379). This study not only fills the theoretical gap in early selection of *E. urophylla* during the full rotation period but also constructs an integrated early selection technology system of “trait screening—model prediction—age determination”. It provides key support for shortening the breeding cycle of *E. urophylla* and achieving precise control of breeding costs and offers important references for early selection research on fast-growing broad-leaved tree species worldwide.

## 1. Introduction

Early selection of forest trees refers to screening based on the correlation between traits at the juvenile stage and the mature stage before trees reach economic maturity. It plays a crucial role in shortening the breeding cycle and accelerating the breeding process [1]. As early as 1976, Wright clearly pointed out that people always expect trees with vigorous growth at the juvenile stage to maintain excellent growth performance at maturity. To realize this expectation, it is essential to screen out potential plants in advance, so early selection cannot be ignored. Early selection is a specific form of correlative selection, based on the correlation analysis of tree traits at the juvenile and mature stages. Currently, the academic community generally believes that early identification and selection of forest trees are feasible and necessary. Among them, the research on early–late trait correlations of forest trees is the most extensive, and the study on early–late growth correlations of forest trees is the main approach to carry out early selection. In recent years, the academic community have systematically conducted research on early selection of growth traits based on early–late correlations for multiple tree species. For coniferous species, studies have been carried out on *Cunninghamia lanceolata* (Lamb.) Hook. [2,3], *Taxus wallichiana* var. chinensis (Pilger) Florin [4], *Pinus elliottii* Engelm. [5], *Larix kaempferi* (Lamb.) Carriere [6], *Pinus tonkinensis* A. Chev. [7], and exotic pines in southwestern Guangxi [8]. For broad-leaved species, studies have been supplemented on *Cyclobalanopsis gilva* (Blume) Oerst. [9] and *Catalpa bungei* C.A. Mey. [10]. Relevant research has even been conducted on herbaceous plants, such as *Nassella pulchra* (Hitchc.) Barkworth and *Bromus diandrus* Roth. [11].

Research on early selection of *Eucalyptus* has been conducted since the late 1990s to the early 2000s. For example, studies were carried out on *Eucalyptus* 12ABL to explore the feasibility and optimal selection age of early selection. Early selection was proven reliable by analyzing the phenotypic and genetic correlation coefficients between early and late growth traits. Furthermore, based on the annual efficiency and optimal selection age of early selection for various growth traits, it was found that early selection for growth traits within 1/5 to 1/3 of the rotation period yields the best results [12]. In addition, Jean-Marc Bouvet et al. conducted a study on retrospective nursery early selection of *Eucalyptus* clones based on growth, morphological, and dry matter criteria. The experiment showed that multi-trait selection combining total growth with growth increment or biomass traits can increase the coefficient of determination. These initial results show that early selection of *Eucalyptus* clones could be used to reject of around 30% of the poorer-growing clones at the mature stage [13]. In recent years, targeted studies have been conducted on *Eucalyptus* cultivars such as *Eucalyptus grandis* × *E. urophylla*, *E. urophylla* × *Eucalyptus tereticornis*, and *Eucalyptus maideni* F. v. Muell. Specifically, studies on *E. grandis* × *E. urophylla* have focused on the effects of seedling morphological characteristics on survival rate, uniformity, and growth during the full short rotation period [14]; research on *E. urophylla* × *E. tereticornis* has emphasized across-rotation genetic analysis and multi-trait selection of a cloned cross [15]; and investigations on *E. maideni* F. v. Muell have focused on the early selection of superior germplasm [16]. All the aforementioned studies have been systematically advanced from different dimensions and achieved remarkable progress, laying a theoretical and technical foundation for the optimization of inter-specific early selection technologies in the *Eucalyptus* genus and their practical application.

However, due to its fast growth rate and excellent fiber quality, *E. urophylla* (Timor mountain gum or Timor white gum) has become one of the core tree species for short-rotation industrial timber plantations in southern China and is widely used in pulpwood and wood-based panel production in South China and Southwest China. Nevertheless, the dynamic correlation rules of its growth traits during the full rotation period have not been clarified, and the research on early selection of clones within this single tree species remains in a weak state. Therefore, in this study, 12 pure *E. urophylla* clones were used as research materials. Focusing on the phenotypic selection of target traits such as fast growth and high growth volume, based on the continuous observation data of growth traits (H, D, and individual V) during the full rotation period from 0.5 to 7.5 years old, the correlation rules of early and late growth traits were clarified through rank correlation and phenotypic and genetic correlation analyses. Combined with the early selection efficiency formula and mathematical modeling, core predictive traits were screened, the optimal selection age was determined, and an early selection model suitable for *E. urophylla* clones was constructed. The results of this study aim to provide a scientific basis for the efficient breeding of *E. urophylla* clones. It not only clarifies the key nodes of early–late correlations of growth traits of *E. urophylla* and fills the theoretical gap in early selection research of this species during the full rotation period, but also provides precise technical support for shortening the breeding cycle and reducing breeding costs of *E. urophylla*. At the same time, it provides a reference for breeding research of other fast-growing broad-leaved tree species [17,18].

## 2. Results

### 2.1. Early–Late Rank Correlation of H, D, and V of E. urophylla Clones

Based on the annual growth data of each trait, the average growth of each clone for each trait was calculated, then the rank of growth of each clone was sorted by trait, and the rank correlation coefficients of the 12 clones for each trait were calculated (Table 1). It can be seen from Table 1 that after 3.5 years, and even more so after 4.5 years, the annual rank correlations of H, D, and V all reached significant or highly significant levels, indicating that early selection of *E. urophylla* clones is possible. After 4.5 years, the correlation coefficients of DBH and volume remained above 0.60 every year, indicating that the annual rank correlations of growth of each clone are relatively stable.

### 2.2. Early–Late Phenotypic and Genetic Correlations of Growth Traits of E. urophylla Clones

Rank correlation is based on the phenotypic values of each clone over the years, which is the result of the combined effect of annual environmental factors and clone heredity. However, growth indicators, as quantitative traits, are highly susceptible to environmental factors. Therefore, rank correlation analysis is not sufficient to serve as the basis for early selection, and it is necessary to calculate the early–late phenotypic and genetic correlation coefficients of each growth trait, which are listed in Table 2. Table 2 also presents the mean values and standard deviations of H, D, and V of *E. urophylla* clones at different ages. The data show that H, D, and V increased steadily with age, showcasing the consistent developmental potential of the clones during the growth period.

The phenotypic and genetic correlation coefficients of each trait showed that in plants aged over 3.5 years, the phenotypic and genetic correlation coefficients of H, D, and V all reached significant or highly significant levels, and the genetic correlations were all greater than the phenotypic correlations. This indicates that the early–late growth correlations of each clone are mainly determined by heredity, and the environmental effect is small.

In addition, the early–late correlation coefficients of H-early versus V-late (estimating late-age volume using early-age H, the same below) and D-early versus V-late basically showed the above characteristics of early–late growth traits. Therefore, early selection based on the field growth performance of tree H, D, and V of forest trees is reliable.

### 2.3. Establishment of Mathematical Models for Early Selection of Growth Traits

Using C.C. Lambeth’s mathematical model for early selection, regression was performed with the natural logarithm of the early–late age ratio (*T_j_*/*T_m_*) as the independent variable and the corresponding genetic correlation coefficient as the dependent variable. The regression equations for each trait are shown in Table 3. From the parameters of each equation, every equation reached a highly significant level, indicating that these equations are applicable and can be used as mathematical models for early selection. The coefficients of determination of D-early versus D-late, V-early versus V-late, and D-early versus V-late were relatively large, with the highest reaching 0.939, indicating an ideal fitting effect of the equations. However, the coefficients of determination for H-early versus H-late and H-early versus V-late are relatively small. Their regression relationships still reach an extremely significant level, but the degree of fitting is relatively low, which may be related to the inconsistent growth rates of different clones in different years. Therefore, selecting D-early versus D-late, V-early versus V-late, and D-early versus V-late as traits for early selection of *E. urophylla* clones will yield more reliable results.

### 2.4. Early Selection Efficiency and Reasonable Selection Age of E. urophylla Clones

Breeders are pursuing timely early selection to reduce breeding costs and achieve economic benefits through breeding as soon as possible. Regarding the efficiency of early selection, when the trait and time are determined, the annual genetic gain of early selection ($\Delta G_{j}$) and the annual genetic gain of mature selection ($\Delta G_{m}$) can be used to evaluate the efficiency of early selection. That is, the level of early selection effect (*E*) is related to $\Delta G_{j}$ and $\Delta G_{m}$ by the following relationship:

$$E=(\Delta G_{j}/T_{m})\cdot (\Delta G_{m}/T_{j})={\hat{r}}_{gjm}\cdot (T_{m}/T_{j}).$$

where ${\hat{r}}_{gjm}$ is the genetic correlation coefficient between early and late traits; (*T_j_*) and (*T_m_*) are the ages of early selection and mature selection, respectively.

When *E* > 1, early selection is superior to mature selection. If the early–late correlation is significant, the early selection intensity is greater than that of late selection, and the early heritability is higher, meaning that the effect of early selection will be better. The value of *T_j_* was calculated based on the magnitude of *E*. In this experiment, the annual efficiency of early selection was calculated with an interval of half a year, and the age with the largest *E* value and significant correlation was the optimal early selection age.

The annual efficiency of early selection for V-early versus V-late was calculated using the early selection efficiency formula (Table 4).

For example, the annual efficiency of using 1-year-old volume to predict volume at a 6-year rotation period was calculated as follows (with a renewal interval of 1 year):

$$E={\hat{r}}_{gjm}\cdot \frac{T_{m}+m}{T_{j}+m}=\left[1.223545+0.697782\ln(\frac{1}{6})\right]\times \frac{6+1}{1+1}=-0.093$$

That is, when clone testing is conducted in the 1-year-old group, the annual efficiency of early selection for volume is −0.093 times that at the rotation period (6 years), which is less than 1. Therefore, the effect of early selection at 1 year old is not ideal. Subsequently, the selection efficiency *E* value increases with the increase in stand age, reaching 1.066 at 2.0 years old (greater than 1), and the maximum *E* value of 1.318 is achieved at 3.5 years old with significant correlation. Finally, with the increase in stand age, the selection efficiency shows a downward trend. Therefore, 3.5 years can be used as the optimal early selection age for *E. urophylla* with a 6-year rotation period. Similarly, according to different cultivation objectives (e.g., the cultivation of medium- and large-diameter timber requires a longer cultivation time), when the rotation periods are set to 8 years, 10 years, and 12 years, the optimal selection ages are 4.5 years old, 5.5 years old, and 6.5 years old, respectively.

In the same way, the maximum annual selection efficiency and optimal selection age for other traits with significant correlation coefficients were calculated and listed in Table 5. The optimal selection age for each trait under different rotation periods ranges from 3.0 to 7.0 years old, accounting for approximately 1/2–2/3 of the rotation period. Early selection conducted at this time has an annual selection efficiency greater than 1, further indicating that early selection is effective.

## 3. Discussion

Rank correlation analysis showed that early selection of *E. urophylla* clones is possible. The phenotypic and genetic correlations are not only consistent in direction, but also their correlation coefficients all reach significant or highly significant levels after the age of 3.5 years. The differences in growth traits of clones are mainly controlled by genetic factors, with small environmental effects. Therefore, early selection based on the growth performance of tree H, DBH, and V of forest trees is reliable. This logic is basically consistent with the idea of Jihyeon Jeon et al. [19], who evaluated the growth and stability of nine poplar clones in riparian afforestation, both eliminating environmental interference to highlight the dominant role of genetic factors in clone traits. Moreover, this result is consistent with the research conclusions of scholars at home and abroad on other tree species. For example, Andrei Caíque et al. [20] found that the early–late correlation coefficients of growth traits at specific ages reached significant levels when studying the early selection of progenies of the timber species *Cordia trichotoma*(Vell.)Arrab. ex Steud. LI Siguang, LI Shuchun, and YE Longtai et al. [21,22,23] conducted studies on *Pinus kesiya* var. *langbianensis* (A.Chev.) Gaussen ex Bui, *Pinus koraiensis* Siebold & Zucc., and *Acacia melanoxylon* R. Br., respectively, and also observed the early–late trait correlation rules and the dominant role of genetic factors in clone selection, further confirming the commonality of early selection among different tree species.

The mathematical models (regression equations) for early selection established based on the early–late correlation coefficients of each growth trait are all applicable. According to the coefficient of determination, using D-early versus D-late, V-early versus V-late, and D-early versus V-late as traits for early selection of *E. urophylla* clones will yield more reliable results. In the studies on *Eucalyptus dunnii* Maiden [24] and *Eucalyptus grandis* W. Hill ex Maiden [25], the validity of regression models was verified through correlation analysis or validation, providing references for the applicability of the model in this study. In practical application, early selection using these three traits can not only judge the growth potential of *E. urophylla* clones earlier and reduce invalid investment in later cultivation, but also maintains a stable selection effect in *E. urophylla* populations in different planting regions, avoiding deviations in selection results caused by environmental differences. At the same time, this selection method based on early–late trait correlations is also consistent with the common idea in forest tree breeding of “early selection and early application to reduce costs”, further indicating the feasibility of this selection scheme in practice.

Based on the individual tree volume performance at 7.5 years of age (TU3: 0.243 m^3^ > TU1: 0.228 m^3^ > TU8: 0.221 m^3^ > TU2: 0.214 m^3^ > TU10: 0.169 m^3^ > TU5: 0.156 m^3^ > MLA: 0.140 m^3^ > TU4: 0.138 m^3^ > TU6: 0.133 m^3^ > U6: 0.129 m^3^ > TU7: 0.108 m^3^ > TU9: 0.060 m^3^), the ranking clearly identifies TU3, TU1, TU8, and TU2 as superior clones, whose volumes were significantly greater than those of the control clones and most other tested clones. These clones all belong to the pure *E. urophylla* species, and their growth advantages primarily originate from genetic factors controlling traits such as fast growth and straight stems. This study further confirms that in clonal breeding, the genotype is the dominant factor determining the early–late correlation of growth traits. Clones such as TU3 and TU1 exhibited stable growth potential, making them promising materials for future adoption and utilization in *Eucalyptus* breeding programs.

According to the annual efficiency of early selection and optimal selection age of each growth trait, the best effect of early selection of growth traits is achieved within 1/2–2/3 of the rotation period. When *E. urophylla* clones are cultivated as pulpwood, the rotation period is generally 6 years, so selection can be conducted at 3.0–4.0 years old, and the selection results are the most reliable. When cultivated as large-diameter timber (with a rotation period of 12 years), selection can be conducted at 5.0–7.0 years of age. This also indicates that the principle of “matching selection age with rotation period” needs to be consistent, and the selection age must be coordinated with the target rotation period to maximize breeding efficiency [26,27,28]. Compared with existing studies, the optimal selection age for *E. urophylla* pulpwood determined in this study is earlier than that of some *Eucalyptus* varieties (e.g., 4.5 years of age mentioned in the study by Jean-Marc Bouvet et al. [29]. This may be due to the faster growth rate of *E. urophylla* and the earlier stabilization time of early growth traits, or it may be related to the sufficient hydrothermal conditions of the experimental site.

Early selection is a key issue in clone breeding, and the possibility and reliability of early selection have been recognized by more and more scholars worldwide. Wen Jing et al. [30]. further confirmed the application value of early selection through multi-trait correlation analysis in the study on early selection efficiency of fiber morphology of *Pinus elliottii* Engelm. It should be clarified that there is basically no difference in individual genetics in clone selection, so early selection of clones is more effective than that of seedling populations. Stepwise selection can also be adopted in early selection; that is, selecting several outstanding clones in the early stage, and then selecting other clones after a period of time. Such continuous screening and continuous application in screening can avoid erroneous selection and missed selection.

## 4. Materials and Methods

### 4.1. Study Site Overview

All three experimental forests in this study were located in the Xiaoze Administrative Region, Daze Town, Xinhui District, Jiangmen City, Guangdong Province (22°34′25″ N, 112°60′12″ E), with an altitude of 45 m. The area belongs to the south subtropical marine monsoon climate zone, with a mean annual temperature of 22.3 °C, an effective accumulated temperature above 10 °C of 7653.0 °C, a mean temperature of the coldest month of 16.7 °C, an extreme minimum temperature of 2.9 °C, and no frost throughout the year. The mean annual rainfall is 1750.4 mm. The forest land is located on low hillslopes with relatively flat terrain and the soil is red earth derived from granite. The afforestation site is the cutover land of *Cunninghamia lanceolata* (Lamb.) Hook. and *Pinus massoniana* Lamb. The soil organic matter content in the 0–50 cm layer is 5.4%, the total N content is 0.31%, the total P content is 0.24%, the total K content is 1.56%, the available N content is 38.43 mg/kg, the available P content is 0.75 mg/kg, the available K content is 9.10 mg/kg, and the pH value (H_2_O) is 5.8. Understory vegetation includes *Echinochloa hainanensis* Chia., *Baeckea frutescens* L., *Melastoma candidum* D. Don, *Rhodomyrtus tomentosa* Hassk., and *Dicranopteris dichotoma* Bernh.

### 4.2. Experimental Materials and Design

A total of 12 pure *E. urophylla* clones were tested (Table 6), among which the U6 clone and MLA clone, which are widely promoted in South China, were used as controls. A randomized complete block design was adopted, with plots of 2 rows × 5 plants and 4 replications. It is arranged in blocks. Hole preparation was conducted with a hole size of 60 cm × 50 cm × 40 cm, and the planting spacing was 4 m × 3 m. During afforestation, 2.5 kg of decomposed chicken manure and 250 g of phosphate fertilizer were applied per hole as base fertilizer.

### 4.3. Methods

The annual growth trait data (H, D, and individual V) of each clone from 0.5 to 7.5 years old in the experiment were used as analysis materials. Rank correlation, as well as early–late phenotypic and genetic correlation analyses, were conducted. A mathematical model for early selection was established, and the early selection efficiency was calculated. For the determination of growth form, H is measured using a Vertex IV-360 infrared hypsometer (manufactured by Haglof Vertex, Sweden), and D is measured for each tree via individual tree inspection with a diameter tape. The formula for calculating the individual V is as follows: *Vol =* 1/30000 *× H × D*^2^*_DBH_* [31].

### 4.4. Statistical Analysis

To test the stability of annual growth of each clone, the Spearman formula [32] was used to calculate the rank correlation coefficient. The formula is as follows:

(1) $$r_{s}=1-\frac{6\sum di^{2}}{n(n^{2}-1)},$$

where *d* is the difference in the rank of the *i*-th pair of clones; *n* is the number of clone pairs; and the *t*-test formula is

(2) $$t=r_{s}(n-2)(1-r_{s}^{2})$$

For early growth prediction, the linear regression model proposed by C.C. Lambeth was used [33], in which the natural logarithm of the early–late age ratio ($T_{j}$/$T_{m}$) was taken as the independent variable, and the corresponding phenotypic or genetic correlation coefficient was taken as the dependent variable to establish an estimation model for early–late correlations of growth traits:

(3) r=a+bLAR

where *a* and *b* are constants; $LAR=\ln(\frac{T_{j}}{T_{m}})$; and *T_j_* and *T_m_* are the number of years required to complete a breeding cycle for late selection and early selection, respectively.

The early selection efficiency was calculated using the early selection efficiency formula proposed by Squillace and Gansel [34]:

(4) $$E={\hat{r}}_{gim}\frac{T_{m}+m}{T_{j}+m}$$

where *E* is the selection intensity; ${\hat{r}}_{gjm}$ is the estimated value of the genetic correlation coefficient between early and late traits; (*T_m_*) and (*T_j_*) are the number of years required to complete a breeding cycle for late selection and early selection, respectively; *m* is the renewal interval.

## 5. Conclusions

The results of the early–late correlation analysis on the growth traits of *E. urophylla* clones during the rotation period showed that early selection is feasible. For *E. urophylla* clones, after 3.5 years of age, the early–late phenotypic and genetic correlation coefficients of their growth traits all reach significant or highly significant levels; the mathematical models (regression equations) for early selection established based on the early–late correlation coefficients of each growth trait are all applicable. According to the coefficient of determination, using D-early versus D-late, V-early versus V-late, and D-early versus V-late as the traits for the early selection of *E. urophylla* clones will yield more reliable results. Based on the annual efficiency of early selection and the optimal selection age for each growth trait, the best effect of early selection of growth traits is achieved within 1/2–2/3 of the rotation period.

## Figures and Tables

**Table 1:** Rank correlation between the early and late growth periods of H, D, and V.

Age	Character	0.5a	1.5a	2.5a	3.5a	4.5a	5.5a	6.5a
1.5a	H	0.413						
2.5a	H	0.532	0.553					
	D		0.923 **					
	V		0.783 **					
3.5a	H	0.692 *	0.448	0.839 **				
	D		0.762 **	0.811 **				
	V		0.734 **	0.818 **				
4.5a	H	0.420	0.294	0.755 **	0.846 **			
	D		0.455	0.441	0.343			
	V		0.315	0.280	0.455			
5.5a	H	0.685 *	0.119	0.483	0.692 *	0.650 *		
	D		0.420	0.385	0.350	0.741 **		
	V		0.252	0.490	0.671 *	0.740 **		
6.5a	H	0.685 *	0.119	0.483	0.692 *	0.650 *	1.000 **	
	D		0.448	0.455	0.420	0.622 *	0.857 **	
	V		0.301	0.399	0.511	0.741 **	0.871 **	
7.5a	H	0.685 *	0.119	0.483	0.692 *	0.650 *	1.000 **	1.000 **
	D		0.448	0.455	0.420	0.622 *	0.857 **	1.000 **
	V		0.301	0.399	0.511	0.741 **	0.871 **	1.000 **

Note: In the table, “a” is the abbreviation of annum (year), denoting the growth age of the plants. * and ** indicate significant differences at the 5% and 1% levels, respectively. The same applies below.

**Table 2:** Phenotypic (P) and genotypic (G) correlation coefficients of H, D, and V growth of *E. urophylla* clones among different ages.

Age	Character	Growth Traits Indicators	0.5a	1.5a	2.5a	3.5a	4.5a	5.5a	6.5a	7.5a
0.5a	H	2.94 ± 0.41		0.992 **	0.293	0.343	0.080	−0.126	−0.126	−0.126
	D									
	V									
1.5a	H	6.74 ± 0.93	0.900 **		0.445	0.609 *	0.145	−0.030	−0.030	−0.030
	D	6.05 ± 0.64			0.634 *	0.423	0.042	−0.028	0.117	0.117
	V	0.01 ± 0.003			0.758 **	0.512	0.140	0.007	0.058	0.058
2.5a	H	8.55 ± 0.70	0.212	0.338		0.689 **	0.312	0.081	0.082	0.082
	D	8.64 ± 0.80		0.455		0.781 **	0.435	0.438	0.470	0.470
	V	0.02 ± 0.005		0.550 *		0.700 **	0.389	0.337	0.393	0.393
3.5a	H	10.06 ± 1.05	0.284	0.569 *	0.702 **		0.878 **	0.712 **	0.713 **	0.713 **
	D	9.70 ± 0.91		0.338	0.776 **		0.823 **	0.852 **	0.824 **	0.823 **
	V	0.03 ± 0.009		0.414	0.715**		0.844 **	0.824 **	0.823 **	0.823 **
4.5a	H	14.25 ± 1.45	0.046	0.142	0.312	0.846 **		0.968 **	0.968 **	0.968 **
	D	12.71 ± 1.54		0.024	0.436	0.802 **		0.992 **	0.980 **	0.980 **
	V	0.08 ± 0.026		0.099	0.385	0.820 **		0.995 **	0.989 **	0.989 **
5.5a	H	15.05 ± 1.37	−0.109	−0.022	0.094	0.682 **	0.935 **		0.985 **	0.987 **
	D	14.16 ± 1.90		−0.029	0.418	0.815 **	0.983 **		0.982 **	0.982 **
	V	0.11 ± 0.036		−0.009	0.328	0.793 **	0.979 **		0.989 **	0.989 **
6.5a	H	15.73 ± 1.43	−0.109	−0.022	0.094	0.682 **	0.935 **	0.958 **		0.997 **
	D	15.88 ± 2.27		0.086	0.426	0.766 **	0.943 **	0.945 **		0.998 **
	V	0.14 ± 0.047		0.025	0.360	0.768 **	0.947 **	0.962 **		0.995 **
7.5a	H	16.55 ± 1.51	−0.109	−0.022	0.094	0.682 **	0.935 **	0.959 **	0.970 **	
	D	16.72 ± 2.39		0.085	0.426	0.766 **	0.943 **	0.945 **	0.969 **	
	V	0.16 ± 0.055		0.024	0.360	0.768 **	0.947 **	0.962 **	0.984 *	

Note: In the table, “a” is the abbreviation of annum (year), denoting the growth age of the plants. Phenotypic correlation coefficients are below the diagonal, and genotypic correlation coefficients are above the diagonal; *R*_(0.05, 12)_ = 0.532, *R*_(0.01, 12)_ = 0.661; * and ** indicate significant differences at the 5% and 1% levels, respectively. The same applies below. The units of the growth traits indicators are as follows: H (m), D (cm), and V (m^3^).

**Table 3:** Regression equations and their parameters for early–late correlation coefficients of growth traits.

Code	Character	Integrated Regress Equation	*R^2^*	*F* Value	*t_a_*	*t_b_*
1	H-early versus H-late	$r_{gjm}=1.039172+0.515757\;LAR$	0.801	20.123 **	6.878 **	4.486 **
2	D-early versus D-late	$r_{gjm}=1.200848+0.728151\;LAR$	0.939	61.044 **	16.434 **	7.813 **
3	V-early versus V-late	$r_{gjm}=1.223545+0.697782\;LAR$	0.929	52.577 **	14.281 **	7.251 **
4	H-early versus V-late	$r_{gjm}=0.881165+0.436114\;LAR$	0.836	25.559 **	7.773 **	5.056 **
5	D-early versus V-late	$r_{gjm}=1.106067+0.625883\;LAR$	0.932	57.352 **	15.399 **	7.511 **

Note: ** indicate highly significant differences at the 1% level.

**Table 4:** Annual efficiency of early selection for V-early versus V-late of *E. urophylla* clones.

Age of Early Selection	Rotation							
	6a		8a		10a		12a	
	${\hat{r}}_{gjm}$	E	${\hat{r}}_{gjm}$	E	${\hat{r}}_{gjm}$	E	${\hat{r}}_{gjm}$	E
1.0a	−0.027	−0.093	−0.227	−1.024	−0.383	−2.107	−0.510	−3.317
1.5a	0.256	0.717	0.055	0.200	−0.100	−0.441	−0.227	−1.183
2.0a	0.457	1.066	0.256	0.769	0.101	0.369	−0.027	−0.116
2.5a	0.613 *	1.225	0.412	1.059	0.256	0.805	0.129	0.479
3.0a	0.740 **	1.295	0.539	1.213	0.383	1.054	0.256	0.833
3.5a	0.847 **	1.318	0.647 *	1.293	0.491	1.200	0.364	1.051
4.0a	0.941 **	1.317	0.740 **	1.332	0.584 *	1.285	0.457	1.188
4.5a	--	--	0.822 **	1.345	0.666 **	1.333	0.539 *	1.274
5.0a	--	--	0.896 **	1.343	0.740 **	1.356	0.613 *	1.327
5.5a	--	--	0.962 **	1.332	0.806 **	1.365	0.679 **	1.358
6.0a	--	--	--	--	0.867 **	1.363	0.740 **	1.374
6.5a	--	--	--	--	0.923 **	1.354	0.796 **	1.379
7.0a	--	--	--	--	--	--	0.847 **	1.377

Note: In the table, “a” is the abbreviation of annum (year), denoting the growth age of the plants. * and ** indicate significant differences at the 5% and 1% levels, respectively.

**Table 5:** Optimal selection age (OSA) and annual selection efficiency (*E*) of different traits.

Character	Rotation							
	6a		8a		10a		12a	
	OSA	*E*	OSA	*E*	OSA	*E*	OSA	*E*
H-early versus H-late	3.0	1.193	4.0	1.227	4.5	1.255	5.5	1.274
D-early versus D-late	4.0	1.271	5.0	1.291	6.0	1.306	7.0	1.317
V-early versus V-late	3.5	1.318	4.5	1.345	5.5	1.365	6.5	1.379
H-early versus V-late	3.0	1.013	4.0	1.042	4.5	1.066	5.0	1.082
D-early versus V-late	3.5	1.196	4.5	1.221	5.5	1.239	6.5	1.252

Note: In the table, “a” is the abbreviation of annum (year), denoting the growth age of the plants.

**Table 6:** Overview of tested clones.

Clone No.	Genotypes	Characteristics	Breeder	Clone No.	Genotypes	Characteristics	Breeder
TU1	U	Fast-growing; straight-stemmed	RITF, CAF	TU7	U	Fast-growing; straight-stemmed	RITF; CAF
TU2	U	Fast-growing; straight-stemmed	RITF, CAF	TU8	U	Fast-growing; straight-stemmed	RITF; CAF
TU3	U	Fast-growing; straight-stemmed	RITF, CAF	TU9	U	Fast-growing; straight-stemmed	RITF; CAF
TU4	U	Fast-growing; straight-stemmed	RITF, CAF	TU10	U	Fast-growing; straight-stemmed	RITF; CAF
TU5	U	Fast-growing; straight-stemmed	RITF, CAF	MLA (CK1)	U	Fast-growing; wind-resistant	Leizhou Forestry Bureau
TU6	U	Fast-growing; straight-stemmed	RITF, CAF	U6 (CK2)	U	Fast-growing; wind-resistant	Zhanjiang Forestry Bureau

Note: U represents *E. urophylla*; RITF, CAF represents the Research Institute of Tropical Forestry, Chinese Academy of Forestry. Control clones are U6 and MLA (commercial clones of *E. urophylla*).

## Data Availability

The original contributions presented in this study are included in the article. Further inquiries can be directed to the corresponding author.
